# The Formation of σ-Hole Bonds: A Physical Interpretation

**DOI:** 10.3390/molecules29030600

**Published:** 2024-01-26

**Authors:** Jane S. Murray

**Affiliations:** Department of Chemistry, University of New Orleans, New Orleans, LA 70148, USA; jane.s.murray@gmail.com

**Keywords:** σ-hole bond formation, iso-density contour envelopes, electrostatic potentials, conceptual experiments at equilibrium and upon approach

## Abstract

This paper discusses two quite different computational experiments relating to the formation of σ-hole bonds A···B. The first involves looking at the complex at equilibrium and finding the contour X of the electronic density which allows the iso-density envelopes of A and B to be nearly touching. This contour increases, becoming closer to the nuclei, as the strength of the interaction increases. The second experiment involves allowing A and B to approach each other, with the aim of finding the distance at which their 0.001 a.u. iso-density envelopes are nearly merging into one envelope. What is found in the second experiment may be somewhat surprising, in that the ratio of the distance between interacting atoms at this nearly merging point—divided by the sum of the van der Waals radii of these atoms—covers a narrow range, typically between 1.2 and 1.3. It is intriguing to note that for the dataset presented, approaching molecules attracted to each other appear to do so unknowing of the strength of their ultimate interaction. This second experiment also supports the notion that one should expect favorable interactions, in some instances, to have close contacts significantly greater than the sums of the van der Waals radii.

## 1. σ-Holes

The widely used term “σ-hole” was introduced quite spontaneously by Tim Clark at a meeting in Prague in September 2005 after a talk by Peter Politzer, which focused on halogen bonding and included surface electrostatic potentials of halogenated methanes [1]. Clark’s short sentence, “It’s the σ-hole!”, was an explanation of why there are positive potentials found on the extensions of some carbon–halogen bonds [2,3]. This led to a paper published in a special issue of this *Molecular Interactions in Biomolecules* (*MIB-II*) meeting [4]; it is the first reference to the “σ-hole”. It did not take long for the search for σ-holes to extend to groups 14 to 16 of the periodic table [5,6,7,8,9,10,11,12,13,14,15], as well as to hydrogen bonding [7]. After eighteen years, the “σ-hole” is cited in the literature for the interactions of aerogens/noble gases [16] and many groups of the transition series [17,18,19,20,21,22,23] with negative sites. This terminology has become so ingrained in the literature that often no citations are even given.

What is a σ-hole? In very simple terms, a σ-hole refers to a diminished electronic density along the extension of a covalent bond to a particular atom. This diminished electronic density often results in a positive electrostatic potential [5,6,7,24] that can interact favorably with negative sites on other molecules, or within the same molecule.

The σ-holes reflect the anisotropy that occurs upon the formation of covalent bonds. Let us talk first about free atoms. Free neutral atoms have electronic densities that are, on average, spherically symmetrical and have positive electrostatic potentials at all points in their surrounding spaces [25]. This is because the concentrated positive nuclear charge dominates over the dispersed electrons [26].

Once two or more atoms combine to form a molecule, the picture changes. Negative regions of electrostatic potential emerge, often associated with lone pairs, π electrons, and strained bonds [27]. For a halogen, where the seventh valence electron forms a covalent bond with its partner, a region of depleted electronic density is found on the extension of that bond. This is the σ-hole [5,6,7]. The same general explanation holds for other groups in the periodic table. The σ-holes often, but not always, have positive potentials associated with them [5,7,24,28,29]. 

It is important to note that the electrostatic potentials on the surfaces of isolated molecules A and B do not reflect the polarization that occurs when they approach each other [30]. This polarization is an intrinsic part of any Coulombic interaction [31,32,33,34,35,36,37,38,39,40,41], as has been recognized since the early use of the electrostatic potential in chemistry [31,32,33,34]. 

In this paper, two “experiments” will be discussed. The first involves changing the electron density contours used to plot the electrostatic potentials of A···B complexes at equilibrium, such that the envelopes surrounding A and B are nearly touching (and are closer to the nuclei [42,43]). The second experiment probes the formation of σ-hole-bonded complexes; in particular, it identifies the distance at which the two 0.001 a.u. envelopes surrounding molecules A and B are soon to merge into one envelope as A and B approach each other. The latter experiment is presented in this paper for the first time.

## 2. Experiment 1: σ-Hole Bonds at Equilibrium

**Figure 1:**
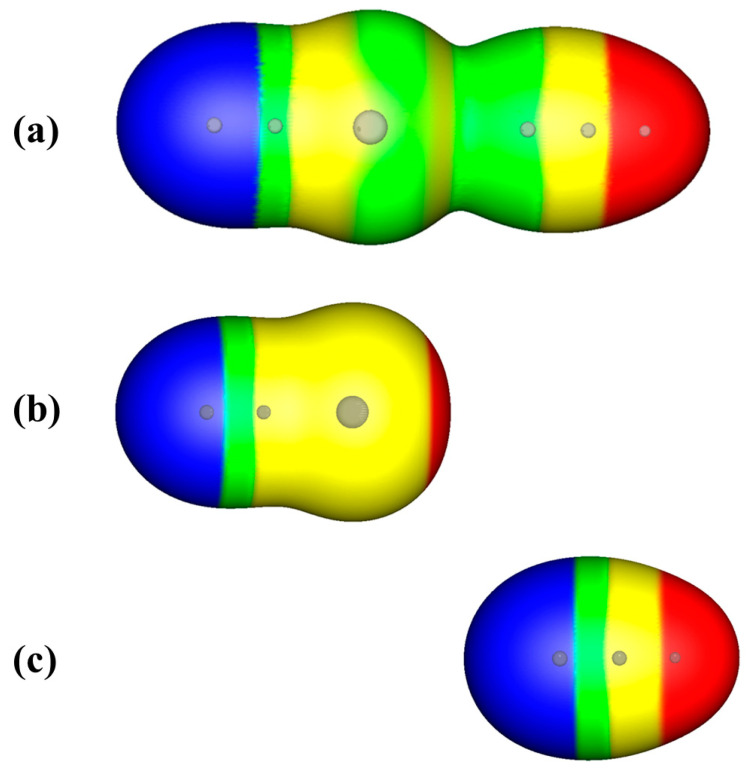
Computed electrostatic potentials on the 0.001 a.u. iso-density surfaces of (**a**) NCBr···NCH at the equilibrium distance between Br and N of 2.97 Å; (**b**) free NCBr; and (**c**) free NCH. Color ranges, in kcal/mol are red, greater than 20; yellow, between 20 and 0; green, between 0 and −10; blue, more negative than 0. Gray spheres show the positions of the atoms within the envelopes.

If one computes the electrostatic potential on the 0.001 a.u. surface of a complex, the driving forces of the interaction cannot be visualized because they are hidden within the envelope [24,42,43]. An example of this is shown in Figure 1a for NCBr···NCH. When one looks at the electrostatic potentials of NCBr and NCH separately, the driving forces of the interaction can be seen. The positive σ-hole on the bromine of NCBr and the negative region associated with the nitrogen lone pair on NCH are shown in Figure 1b,c. 

The electrostatic potentials in Figure 1 are all plotted on the 0.001 a.u. contour of the electronic density, following Bader et al. [44]. This contour contains about 99% of the electronic density surrounding a molecule; the distances from the envelope to the nuclei of atoms are slightly beyond the van der Waals radii of most atoms. This contour is thus a useful one upon which to plot the electrostatic potential for the elucidation of possible future interactions [24].

All calculations have been carried out at the M06-2X/6-311G (3df,2p) level, using Gaussian 09 (Revision A.1) or Gaussian 16 (Version 1.1) [45,46], while surface electrostatic potentials have been computed using the Wave Function Analysis—Surface Analysis Suite (WFA-SAS) [47]. The M06-2X functional was designed specifically for noncovalent interactions [48,49]. The WFA-SAS code allows one to choose the contours of the electronic density upon which to plot the electrostatic potential. This ability will be utilized in this paper, as it has recently been used in studies of intramolecular interactions [50,51].

**Figure 2:**
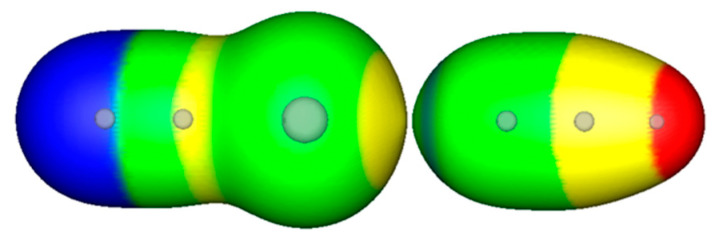
Computed electrostatic potentials on the 0.014 a.u. iso-density surface of NCBr···NCH at the equilibrium distance between Br and N of 2.97 Å. Color ranges, in kcal/mol are red, greater than 100; yellow, between 50 and 0; green, between 50 and 0; blue, negative. Gray spheres show the positions of the atoms within the envelopes.

Table 1 shows computational data for fifteen complexes A···B [42,43]. It includes the interaction energies for the formation of the complexes, the equilibrium distances between A and B, and the equilibrium distances between A and B divided by the sums of the van der Waals radii [52] for the interacting atoms. The fifth column in Table 1 gives the contour X of the electronic density that allows the electron density envelopes of A and B to be nearly touching [42,43]. Figure 2 shows an example of this for NCBr···NCH, where the nearly-touching contour is 0.014 a.u. for this complex.

It is noteworthy to compare the plots of the electrostatic potentials on the 0.001 a.u. surfaces of NCBr and NCH, as shown separately in Figure 1b,c, and that of the nearly-touching contour X, the 0.014 a.u., for the complex NCBr···NCH in Figure 2. For the free NCBr and NCH molecules in Figure 1b,c, the curvature of the surfaces around the bromine of NCBr and the nitrogen of NCH are smooth. As shown in Figure 2, a close examination of the nearly touching envelopes of NCBr and NCH for the complex NCBr···NCH, at the 0.014 a.u. contour of the electronic density, reveals the polarization of the electron density that is occurring. The shape of the envelope around the bromine of NCBr is slightly pointed toward its interaction partner, NCH. Likewise, the shape of the envelope around the nitrogen of NCH is also slightly pointed toward the bromine of NCBr. 

In Table 1, as might be expected, one can see that as the interaction energies become more negative, the equilibrium distances of the interactions divided by the sum of the van der Waals radii generally become smaller, and the contours X tend to be larger (closer to the nuclei). This has been noted in earlier work [42,43]. This approach has allowed us to find the relative impenetrable volumes for interacting molecules in a particular complex and to predict the “absolute” impenetrable volumes of molecules to be about 25% of their 0.001 a.u. volumes [42]. For the complex NCBr···NCH shown in Figure 1 and Figure 2, and as discussed, the volume of this complex at the 0.014 a.u. contour is ~38% of the 0.001 a.u. volume, approaching the limit of 25% found to define “absolute” impenetrable volumes [42]. 

It has been documented in many papers that the magnitudes of the most positive and most negative potentials (V_S,max_ and V_S,min_) of the partners in the complexes prior to interaction relate to the strengths of these interactions [5,7,11,24]. For this reason, these values are not listed in Table 1.

## 3. Experiment 2: From Infinity until Nearly Merging Electronic Densities in σ-Hole Bonding

The objective in this section is to introduce “experiment 2”, which aims to observe the approach of partners A and B leading to their ultimate equilibrium interaction, and to identify the distance at which the separate 0.001 a.u. iso-density envelopes nearly merge into one. In Table 2, the same fifteen interactions are listed as in Table 1; the last column of this table gives the distances between the interacting portions of A and B at this “nearly merging” point, divided by the sums of the van der Waals radii.

This experiment has been discussed recently only for the first interaction, Ar···Ar, in Table 2. At distances between the Ar atoms of 10 Å, the argon atoms show no interaction; their surface electrostatic potentials are isotropic and positive [26]. As they approach, such that the distance between the Ar nuclei is 5 Å, the Ar atoms begin to interact, and the closest parts of their 0.001 a.u. envelopes are less positive than the outer portions. Why is this so? This is a result of the slight polarization of each atom’s electron density toward the other. At the equilibrium distance of 3.758 Å, there is only one envelope encompassing the two argon atoms. The electrostatic potential on the 0.001 a.u. surface at this point between the two argon atoms is now more positive than before, even though there is a buildup of electronic density between the argon atoms. This is because of the positive nuclear charges on the now closer argon nuclei. This and other examples point out the importance of recognizing that the electrostatic potential does not always track the electronic density [26].

**Figure 3:**
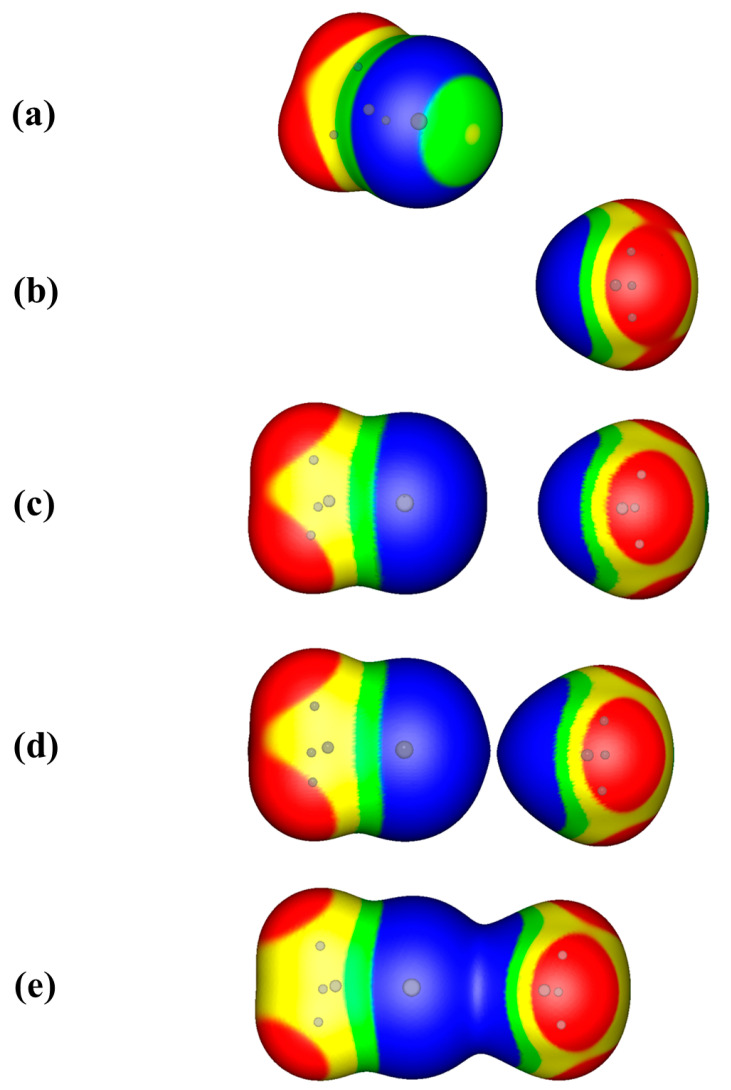
Computed electrostatic potentials on the 0.001 a.u. iso-density surfaces of (**a**) free methyl chloride (H_3_C-Cl); (**b**) free ammonia (NH_3_); (**c**) H_3_C-Cl and NH_3_ approaching each other at 2 Å beyond their equilibrium distance of 3.10 Å; (**d**) H_3_C-Cl and NH_3_ approaching each other at 4.26 Å, near the merging point of electronic densities; and (**e**) H_3_C-Cl···NH_3_ at equilibrium. Color ranges, in kcal/mol are red, greater than 10; yellow, between 10 and 0; green, between 0 and −10; blue, more negative than 0. Gray spheres show the positions of the atoms within the envelopes.

There are fifteen complexes in Table 1 and Table 2. The first, Ar···Ar, discussed above, can be classified as a counter-intuitive interaction [28], which is when both partners in an interaction have either positive electrostatic potentials or negative electrostatic potentials as they approach each other. The fourth interaction in Table 1 and Table 2 is between H_3_C-Cl and NH_3_. This interaction can also be classified as counter-intuitive: as can be seen in Figure 3a, the electrostatic potential on the extension of the carbon–chlorine bond is very close to neutral. Depending on the method/basis set combination that one chooses, the chlorine V_S,max_ is either very slightly negative or very slightly positive [28]. Yet, there is an attractive interaction between the chlorine of H_3_C-Cl and the negative nitrogen of NH_3_.

Figure 3b shows the electrostatic potential on the 0.001 a.u. surface of NH_3_. The lone pair electrostatic potential of NH_3_ is more negative than that of NCH, as noted previously [7,11,28]. Figure 3c shows the approaching H_3_C-Cl and NH_3_ molecules, at a distance of 5.10 Å, just 2 Å beyond the equilibrium distance of the complex (3.10 Å). The chlorine of H_3_C-Cl is now totally negative, as is the nitrogen of NH_3_. The curvatures around the chlorine of H_3_C-Cl and the nitrogen of NH_3_ are smooth, with no clear signs of ensuing interaction. When we move closer, with a distance of 4.257 Å between the chlorine and the nitrogen, as shown in Figure 3d, the envelopes are close to merging into one. Here, one can see that the envelopes surrounding the interacting atoms are pointed toward each other, showing the polarization of their electronic densities as H_3_C-Cl and NH_3_ approach each other. Finally, in Figure 3e, at the equilibrium distance of the complex, there is only one envelope. The driving forces of the interaction are hidden within, as we have seen in Figure 1a for the NCBr···NCH interaction.

**Figure 4:**
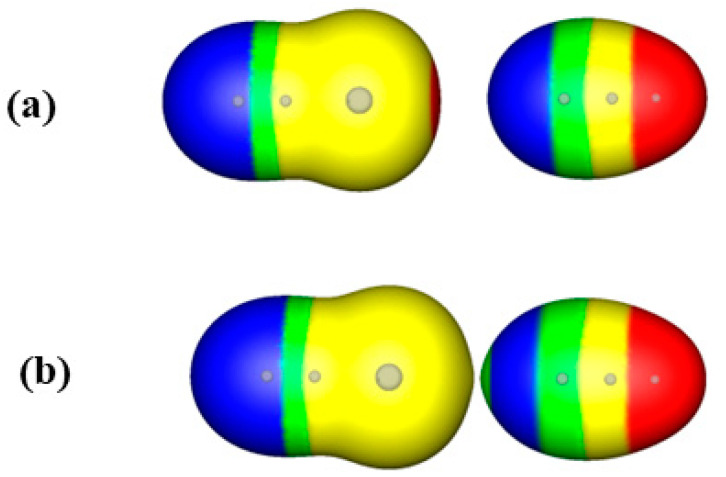
Computed electrostatic potentials on the 0.001 a.u. iso-density surfaces of (**a**) NCBr and NCH approaching each other at 2 Å beyond their equilibrium distance of 2.97 Å; and (**b**) NCBr and NCH approaching each other at 4.17 Å, near the merging point of electronic densities; Color ranges, in kcal/mol are red, greater than 20; yellow, between 20 and 0; green, between 0 and −10; blue, more negative than 0. Gray spheres show the positions of the atoms within the envelopes.

Let us look now at the approach of NCBr and NCH as the two molecules get closer to the equilibrium distance of 2.97 Å for the NCBr···NCH interaction. Figure 4a shows the approaching molecules at a distance of 4.97 Å, 2 Å beyond the equilibrium distance. At this separation, the two molecules have separate envelopes and look very much like the plots of the isolated NCBr and NCH molecules shown in Figure 1b,c. As the molecules move closer to each other, to the distance of 4.17 Å, the separate envelopes are preparing to merge into one (see Figure 4b). As shown in Figure 3d for the approach of methyl chloride and ammonia, it can be seen that the envelopes of the interacting atoms (Br and N) are pointed toward each other, showing the polarization of their electronic densities.

Looking more closely now at Table 2, one striking feature to note is that the ratio of the distance between A and B at the nearly-merging point of the 0.001 a.u. envelopes to the sum of the van der Waals radii of the interacting atoms covers a small range, from 1.14 to 1.33. This is quite different from the range of the equilibrium distance between A and B divided by the sum of the van der Waals radii, which ranges from 1.0 to 0.7, and contours X of the nearly-touching points for the same molecules, which range from 0.0015 a.u. to 0.056 a.u., as shown in Table 1. The correlation coefficients (R) for the relationships between interaction energy and these quantities are 0.893 and 0.947, respectively.

What are the ratios in the fourth column of Table 2 suggesting? The overall lack of correlation between the interaction energies and ratios of the distances to the nearly merging points/sums of van der Waals radii suggests that the approaching molecules have little notion ahead of time as to what the strength of their ultimate interaction will be. This is yet to be determined. The second comment is that the merging of the 0.001 a.u. envelopes of A and B begins at between 1.2 and 1.3 times the sum of the van der Waals radii. We will return to this in the next section.

## 4. Discussion and Concluding Remarks

In this paper, two “experiments” relating to the formation of intermolecular complexes A···B are presented. The first involves changing the contours defining the iso-density envelopes of the electronic density of intermolecular complexes from the 0.001 a.u. to what is referred to as the nearly-touching contour X, at the equilibrium distances of the complexes. The second focuses on observing the approach of A and B at the 0.001 a.u. contours of the electronic density. The first experiment has been discussed earlier [42,43] and has recently been extended to intramolecular interactions [50,51]. The second experiment is being presented for the first time in this work.

What is found in the first experiment is that at equilibrium, the nearly-touching contour X becomes larger (closer to the nuclei) as the interaction energy becomes more negative and as the equilibrium distances divided by the sums of the van der Waals radii become smaller. These general tendencies have been pointed out in earlier work [42,43].

Findings from the second experiment were somewhat unexpected. The ratio of the nearly-merging point distance to the sum of the van der Waals radii of the interacting atoms does not show considerable variation. It is seen in Table 2 that these ratios range from 1.20 to 1.24 for interactions involving NCH and OCO as the negative sites, while those involving ammonia as the negative site range from 1.27 to 1.31. This slightly larger ratio for the complexes involving ammonia can conceivably be attributed to the negative region of the nitrogen of ammonia being more negative than that of NCH [7,28], such that an approaching species feels the presence of NH_3_ before that of NCH.

The data in Table 2 are in accordance with recent comments about the use and misuse of van der Waals radii [52,53,54,55,56]. It is often beyond the sums of these distances that interactions begin. Experiment 2 supports this. It is also intriguing to note that the ratios in the last column of Table 2 do not clearly suggest the strengths of the ultimate interactions, except perhaps for the weakest, Ar···Ar.

Supporting the use of the M06-2X/6-311G(3df,2p) method/basis set combination, natural bond orbital (NBO) charges [34] have been computed for three of the interactions in Table 1 and Table 2. For H_3_C-Cl···NH_3_, NCBr···NCH, and Cl-Cl···NH_3_, the natural charges at the nearly-merging point distances of the 0.001 a.u. envelopes are slightly less positive than for the halogens at the equilibrium distances for the complexes, as expected. Also, the natural charges for the nitrogen of ammonia are more negative than those for the nitrogen of HCN. It is important, however, to recognize that charges on atoms that have both positive and negative electrostatic potentials on their surfaces, such as many halogens [2,3,4,5,7,11,24], cannot correctly predict halogen bonding and other σ-hole interactions [37,38,41].

It is interesting to point out that both experiments 1 and 2 show the polarization of the electronic densities of partners A and B. Experiment 1 shows this at the nearly-touching contour X at the equilibrium geometry (e.g., Figure 2). Meanwhile experiment 2 shows this at the nearly-merging point distances of the electron density envelopes on the 0.001 a.u. contours of the electronic density, as partners A and B approach (Figure 3d and Figure 4b). The second experiment shows that the interactions between A and B begin beyond the van der Waals radii of the closest atoms and supports the notion that distances greater than 1.2 times the van der Waals radii may indicate favorable interactions.

The purpose of this paper has been to consider two different yet complementary conceptual experiments and to discuss them together, as has been achieved. This work can certainly be expanded in the future to include larger datasets and differing methodologies. Both experiments allow visualization of the polarization that occurs in forming noncovalent σ-hole interactions, but from two different perspectives.

## Figures and Tables

**Table 1:** Computed interaction energies, equilibrium distances between interacting atoms of A and B, and other properties for fifteen bimolecular complexes A···B ^a,b,c^.

Complex	ΔE (kcal/mol)	Equilibrium Distance A···B (Å)	Eq. Dist. A···B/Sum of vdW Radii ^a^	Contour X (a.u.) of Nearly-Touching Point ^b^
Ar···Ar	−0.2	3.76	1.00	0.0015
H_3_C-Cl···OCO	−0.5	3.26	1.00	0.0046
NCF···NCH	−1.5	2.98	0.99	0.006
H_3_C-Cl···NH_3_	−1.9	3.10	0.94	0.011
NCF···NH_3_	−1.9	2.97	0.98	0.0081
NCH···OCO	−2.2	2.24	0.82	0.011
NCCl···NCH	−3.7	2.91	0.88	0.013
NCBr···NCH	−4.6	2.97	0.87	0.014
NCH···NCH	−4.9	2.22	0.84	0.015
NC(F)Se···NCH ^c^	−5.3	2.94	0.85	0.015
FBr···NCH	−7.1	2.64	0.78	0.026
ClCl···NH_3_	−7.9	2.60	0.79	0.0277
NCH···NH_3_	−8.5	2.10	0.76	0.0222
NCH···N(CH_3_)_3_	−13.1	2.01	0.73	0.029
FBr···NH_3_	−15.6	2.38	0.70	0.056

^a^ van der Waals radii are from Ref. [52]. ^b^ Some data is taken from Refs. [42,43]. ^c^ Computed for the present paper.

**Table 2:** Computed interaction energies and other properties for fifteen bimolecular complexes A···B.

Complex	ΔE (kcal/mol)	Eq. Dist. A···B/Sum of vdW Radii ^a^	Dist. of A···B at Merging Point of 0.001 a.u. Envelopes/Sum of vdW Radii ^a^
Ar···Ar	−0.2	1.00	1.14
H_3_C-Cl···OCO	−0.5	1.00	1.20
NCF···NCH	−1.5	0.99	1.22
H_3_C-Cl···NH_3_	−1.9	0.94	1.29
NCF···NH_3_	−1.9	0.98	1.26
NCH···OCO	−2.2	0.82	1.20
NCCl···NCH	−3.7	0.88	1.23
NCBr···NCH	−4.6	0.87	1.23
NCH···NCH	−4.9	0.84	1.24
NC(F)Se···NCH ^b^	−5.3	0.85	1.23
FBr···NCH	−7.1	0.78	1.23
ClCl···NH_3_	−7.9	0.79	1.28
NCH···NH_3_	−8.5	0.76	1.31
NCH···N(CH_3_)_3_	−13.1	0.73	1.33
FBr···NH_3_	−15.6	0.70	1.27

^a^ van der Waals radii are from Ref. [52]. ^b^ Computed for the present paper.

## Data Availability

The data presented in this study are available on request from the corresponding author.
